# Leek or Garlic? A Chemical Evaluation of Elephant Garlic Volatiles

**DOI:** 10.3390/molecules25092082

**Published:** 2020-04-29

**Authors:** Roberta Ascrizzi, Guido Flamini

**Affiliations:** 1 Dipartimento di Farmacia, Università di Pisa, Via Bonanno 6, 56126 Pisa, Italy; guido.flamini@unipi.it; 2 Centro Interdipartimentale di Ricerca “Nutraceutica e Alimentazione per la Salute”, Università di Pisa, 56126 Pisa, Italy

**Keywords:** *Allium ampeloprasum* var. *holmense* (Mill.) Asch. et Graebn., big tex garlic, garlic, great headed garlic, headspace, leek, Tahiti garlic

## Abstract

“Aglione della Valdichiana” is listed among the Traditional Agronomic and Edible Products of Italy, as it is a typical product of the Chiana Valley (Tuscany, Italy). It is also known as “elephant garlic”, due to the dimension of its cloves, and, other than in the Italian Mediterranean area, its presence is also reported in North Africa and Southwest Asia. The current botanical classification identifies it as a leek variety (*Allium ampeloprasum* L.), although its appearance, except for its larger dimensions, resembles that of garlic. In the present study, the spontaneous volatile emission of whole and cut cloves of “Aglione della Valdichiana” (elephant garlic), garlic, and leek has been profiled by headspace solid phase micro-extraction. The results have been subjected to statistical analyses (analysis of variance, hierarchical cluster, and principal component analysis) to assess whether the chemical profile confirmed the botanical proximity of elephant garlic and leek, rather than garlic. The phytochemical volatiles evaluation indicated a higher proximity of elephant garlic to garlic, rather than leek, at least for the Chiana Valley specimen analyzed in this study.

## 1. Introduction

With a physical appearance like garlic, but botanically and genetically characterized as a leek variety, the ‘Aglione della Valdichiana’ is a species of *Allium* (Amaryllidaceae family) typical of the Italian Chiana Valley, which lies between the Tuscan provinces of Arezzo and Siena, and the Umbrian provinces of Terni and Perugia. It has been included in the list of Traditional Agronomic and Edible Products of Italy by the Italian Ministry of Agriculture, Food and Forests (Update no. 176 of 14 July 2017, Annex I, Section ‘Raw or Transformed Vegetable Products’ of the Tuscany Region). Commonly referred to as ‘elephant garlic’, ‘giant head garlic’, or ‘Tahiti garlic’, its botanical name is reported as *Allium ampeloprasum* var. ‘Holmense’ (Mill.) Asch. et Graebn. [1], but the latter is reported as a synonymic form of *Allium ampeloprasum* L. on The Plant List and International Plant Name Index (IPNI) databases (accessed November, 2019). The dilemma underlying elephant garlic botanical classification is yet unresolved: for the species *ampeloprasum*¸ a complex and large number of varieties and synonyms is reported, which led to the proposal of the existence of an “*A. ampleloprasum* complex”, as the boundaries of this species are difficult to assess [2]. Being a wild species, *A. ampeloprasum* is extremely variable, and, due to its economic importance, it has been intensely cultivated and selected in many countries, especially in Europe [3]. Elephant garlic is considered a cultigen, originated as a hybrid of the wild *A.* a*mpeloprasum* and the local varieties of elephant garlic, which co-exist in all the Mediterranean area [4,5]. This species is characterized by very large cloves, which reach up to 70–80 g, and an oversized bulb that can reach 500 g [1]. The flowers are gathered in large heads and are usually seedless (sterile) [1]. The reproduction is achieved using its cloves, since it does not produce vital seeds as it is self-incompatible; the seeding is performed from October to December, and the harvest takes place between June and July [4]. It is the main ingredient of a Tuscan regional pasta recipe (‘Pici all’Aglione’); it can be used instead of garlic, as its flavour is milder and more pleasant. ‘Aglione’, though, is an endangered species, as it has never been produced in large amounts. Recently, the production accounts for around 2000 bulbs per year, mainly produced for self-consumption: for this reason, it is included in the list of protected species under the Slow Food Presidia [6]. It is also listed in the Register of European Union trademarks as ‘Aglione di Valdichiana’ (registered 19 May, 2016 no. 014955471) [7].

As a species belonging to *Allium* genus, it is rich in sulfur-containing compounds, to which the health benefits of garlic-related species are attributed [8]. Some of those sulfur-compounds are part of the volatile emission of these species, of which they define the typical aroma perceived during their consumption as foods.

The present study aimed at analysing the emitted volatiles of whole and cut elephant garlic cloves compared to samples of garlic (*Allium sativum* L.) and leek (*Allium ampeloprasum* L.) in order to assess the chemotaxonomical classification of this species as closer to garlic or leek.

## 2. Results

### 2.1. Headspace Compositions

Overall, 69 volatile organic compounds (VOCs) were detected among all the analyzed headspaces (HSs), whose compositions were identified in percentages ranging from 96.3% to 100%. The identified VOCs in all the samples are reported in Table 1. Sulfide compounds were the most abundant chemical class in all the samples, especially in the cut ones, in which they represented the only detected chemical group. Diallyl disulfide was found as the most abundant compound in the HSs of elephant garlic and garlic samples: in the former, its relative composition reached 38.0% and 61.4% in the whole and cut samples, respectively; in the latter, the whole and the cut sample HSs were dominated by this compound, as it amounted up to 83.4% and 75.8%, respectively. The whole elephant garlic HS exhibited 1-allyl-2-isopropyl disulfane as the second most abundant compound: its presence in this sample was statistically different compared to its cut counterpart, where its relative concentration was almost halved. In the sliced sample, indeed, methyl-2-propenyl disulfide was found as the second most relevant compound. Dipropyl disulfide, instead, dominated the HSs of the leek samples, with relative abundances of over 67% in both the whole and the cut forms. (*E*)-1-(Prop-1-en-1-yl)-2-propyl disulfane followed in the leek samples, where it amounted for 11.3% and 7.8% of the whole and cut samples, respectively. In all of the whole samples, the non-sulfur non-terpene compounds were detected as the second most abundant chemical class of volatiles: *n*-hexadecane was the most relevant in both the garlic and elephant garlic HSs (5.5% and 10.8%, respectively), whilst leek exhibited relative abundances over 1% of isopropyl tetradecanoate. Oxygenated monoterpenes were detected in the HSs of the whole cloves of garlic and elephant garlic with relative abundances over 3.0%: for the former, camphor was the most abundant among the compounds of this class, while 1,8-cineole and α-terpinyl acetate were the most relevant ones in the latter.

As reported in Table 1, with the exception of (*E*)-1-(prop-1-en-1-yl)-2-propyl disulfane and dipropyl trisulfide, a significant interaction between the analyzed species and whether it was analyzed as whole or cut was evidenced for all the major compounds (evidenced in bold in Table 1).

### 2.2. Statistical Analysis

**Figure 1:**
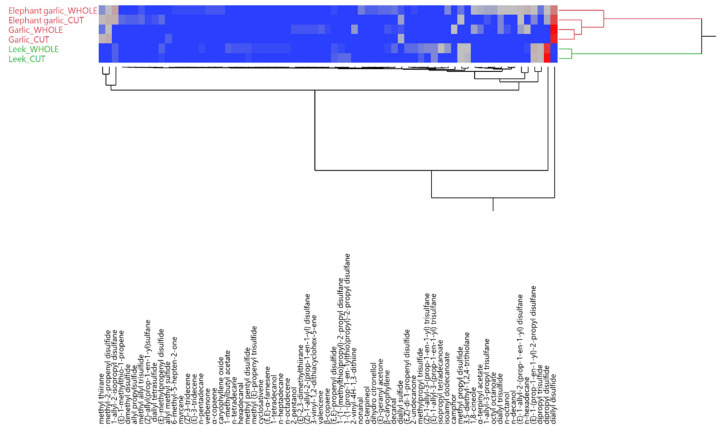
Two-way dendrogram of the hierarchical cluster analysis (HCA) performed on the complete compositions of the headspaces of both whole and cut samples of elephant garlic (*Allium ampeloprasum* var. *holmense* (Mill.) Asch. et Graebn), garlic (*A. sativum* L.) and leek (*A. ampeloprasum* L.).

The hierarchical cluster analysis (HCA) evidenced a first general classification of the samples in two main groups, as shown in the two-way dendrogram in Figure 1. The first cluster (red) comprised the headspaces of both the whole and the cut forms of garlic and elephant garlic, while the second (green) was only composed of the two leek headspaces. In the red cluster, however, the cut cloves of elephant garlic were closer to the garlic headspaces, compared to the whole sample. The clusterization of the detected compounds evidenced a first, main grouping driven by the presence of diallyl sulfide, constituting a cluster on its own. Among all the other compounds, a first sub-grouping was obtained for dipropyl disulfide vs. all the other constituents. Going further, methyl thiirane, methyl2-propenyl disulfide and 1-allyl-2-isopropyl disulfane were clustered by themselves, compared to all the other detected volatiles, whose relative quantities induced lower degrees of differentiation in the dendrogram.

**Figure 2:**
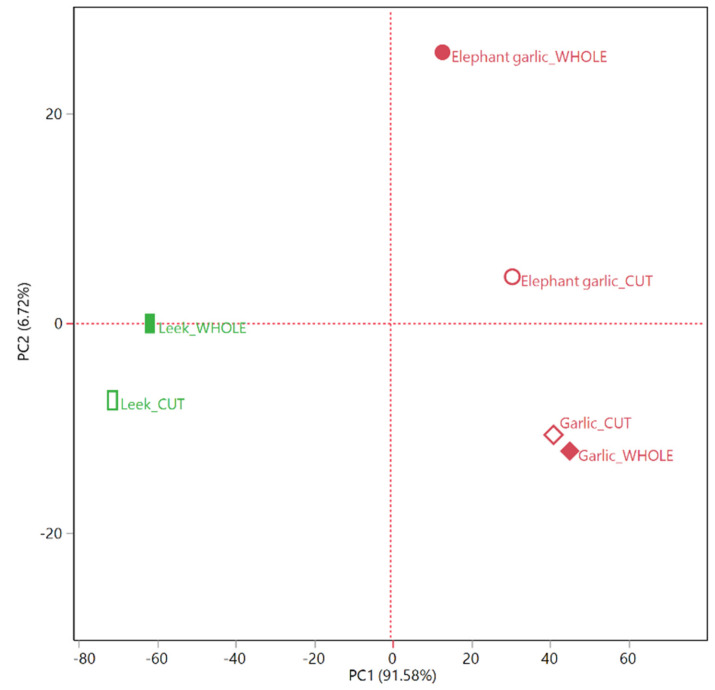
Score plot of the principal component analysis (PCA) performed on the complete compositions of both whole and cut samples of elephant garlic (*Allium ampeloprasum* var. *holmense* (Mill.) Asch. et Graebn), garlic (*A. sativum* L.) and leek (*A. ampeloprasum* L.).

The principal component analysis (PCA) plot (Figure 2), with a total studied covariance of over 98%, confirmed this first distribution of the samples in two groups: both the leek samples were positioned in the left quadrants side (PC1 < 0) of the plot, while the garlic and elephant garlic HSs were distributed in the right quadrants (PC1 > 0). The elephant garlic samples, though, were both plotted in the upper quadrant (PC2 > 0), whilst the garlic ones were both positioned in the lower quadrant (PC2 < 0).

**Figure 3:**
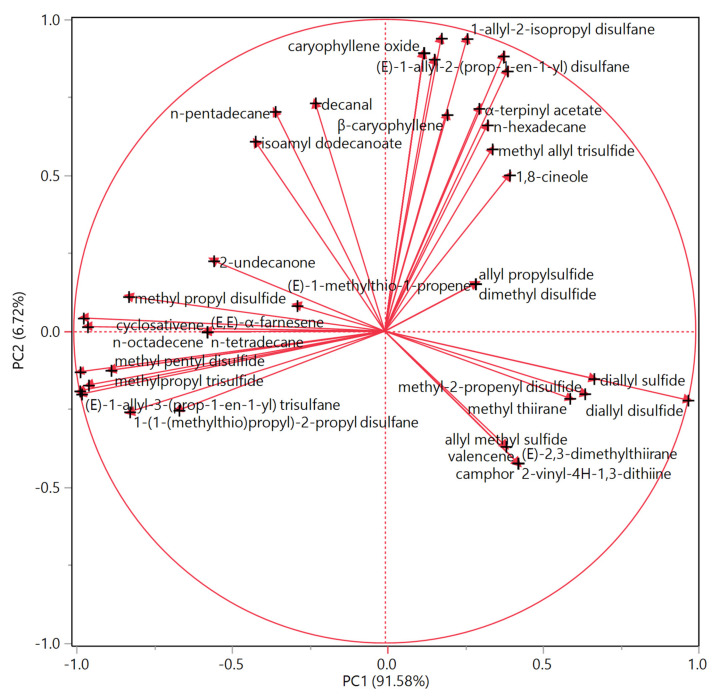
Loadings plot of the principal component analysis (PCA) performed on the complete compositions of both whole and cut samples of elephant garlic (*Allium ampeloprasum* var. *holmense* (Mill.) Asch. et Graebn), garlic (*A. sativum* L.), and leek (*A. ampeloprasum* L.).

The loadings plot (Figure 3) for this PCA showed that the score plot distribution was mainly due to the most abundant compound for each sample. The vectors of dipropyl disulfide and (*E*)-1-(prop-1-en-1-yl)-2-propyl disulfane, both abundant in leek HSs, lie in this lower left quadrant, thus representing the main reason for the positioning of the leek samples in this area of the plot. The garlic samples were plotted on the bottom right quadrant, given their statistically significant higher relative content in diallyl disulfide, whose vector was oriented in this quadrant (Figure 3). Elephant garlic samples were still plotted on the right side of the score plot: diallyl disulfide was largely represented in these samples, as well. Their positioning in the score plot, though, was in the upper right quadrant, as they were also rich in 1-allyl-2-isopropyl disulfane (both) and *n*-hexadecane (only the whole sample), whose vectors pointed in this quadrant.

## 3. Discussion

The presence of sulfide compounds in the cut form of each species was statistically more relevant compared to their whole counterparts: the slicing of the cloves triggers the activity of the alliinase enzyme, causing the breakdown of alliin into allicin, from which all the detected sulfide compounds are originated by decomposition [9]. The predominance of diallyl disulfide in the elephant garlic headspace is in accordance with a previous report on an American specimen [10]. Methyl allyl trisulfide and diallyl tetrasulfide, followed by cyclopentanethiol and diallyl sulfide, were reported as the most abundant organosulfur compounds detected in methanol extracts of an Iranian specimen of *Allium ampeloprasum* var. ‘*Holmense*’ [11]. These differences, however, might be due to the different geographical area of provenience, as well as to the different type of performed analysis.

A higher proximity among the elephant garlic, especially of the headspace of its cut cloves, and garlic samples was evidenced in both the hierarchical cluster (Figure 1) and principal component (Figure 2) analyses, thus indicating a closer chemical similarity between these two, compared to the leek samples. These findings are, thus, conflicting with the present botanical classification of all the elephant garlics (including “Aglione della Valdichiana”) as a leek variety (as *Allium ampeloprasum* L.).

The higher chemical similarity between elephant garlic and garlic, compared to the former and leek, is particularly evident in the absence of allyl groups in leek (with the exception of 1-allyl-2-isopropyl disulfane, found in as low relative concentration as 0.3% in both whole and cut leek samples), while their presence is relevant in the two other species (i.e., diallyl disulfide and (*E*)-1-allyl-2-(prop-1-en-1-yl) disulfane). This finding is in accordance with several published studies [12,13,14]. Moreover, as reported in the literature, 1-propenyl groups were more represented in the volatile emission of leek, but in whole and cut form; (*E*)-1-(prop-1-en-1-yl)-2-propyl disulfane was the second most abundant volatile in leek emissions, while its relative abundance was lower than 3% in elephant garlic and it was not detected in garlic [12]. The need to consider elephant garlic as a separated species from leek was also reported by Hirschegger et al. (2010) [3]. Their published molecular phylogeny study, indeed, distributed these two species in different sub-clades based on the chloroplast analysis, thus differentiating the giant-headed garlic from the other varieties attributed to the *ampeloprasum* species [3].

Its identity as a stand-alone species, also compared to garlic, might be further supported by their quite different nutritional and mineral profiles. A higher crude protein content is reported for elephant garlic, while crude fibers are more abundant in garlic and minerals are more concentrated in garlic, although both species show the same qualitative composition, as well as heavy metals [15]. Even more notably, Cai et al. (1994) [16] detected no after-cut emission of volatile organoselenium compounds for elephant garlic, while they were emitted by garlic, and Lu et al. (2011) [17] evidenced the significantly lower antioxidant power of a 70% methanol extract of elephant garlic compared to the analyzed garlics from four different regions. Further confirmation comes from Hirschegger et al. (2010) [3] molecular phylogeny analysis, which evidenced that, although frequently used as a garlic substitute, only a distant relationship was revealed between the two species. 

The results of the present study, confirming the trends reported in the published literature, seemed to point out the need for a re-evaluation of the existing classification, addressing the possibility of classifying the “Aglione della Valdichiana” as a garlic variety or, at least, as an ecotype, if not as a stand-alone species by itself.

## 4. Materials and Methods 

### 4.1. Plant Material

All the samples were grown in Torrita di Siena (SI), in the Chiana Valley of Tuscany, Italy (43°10′20 N, 11°47′1 E), by a local producer. The specimens have been deposited and registered in the *herbarium* of the Botanic Garden of Pisa with the following voucher numbers: Erbario Generale-N.A. 1049 *Allium sativum* L.: 031415; Erbario Generale-N.A. 1049 *Allium ampeloprasum* L.: 031416; Erbario Generale-N.A. 1049 *Allium ampeloprasum* var. *holmense* Asch. & Graebn.: 031417.

### 4.2. Head-Space Solid Phase Micro-Extraction (HS-SPME)

The headspace spontaneous volatile emissions of the whole and cut forms of the three species were sampled by HS-SPME. Triplicates were performed for each sample. For each replica, the equilibration was performed at room temperature for 30 min for all the samples prior to the volatile adsorption. A Supelco SPME (Solid Phase Micro-Extraction) device coated with polydimethylsiloxane (PDMS, 100 μm) was used (Supelco, Bellefonte, PA, USA), preconditioned according to the manufacturer instructions, for all the analyses. Sampling was accomplished in an air-conditioned room (22 ± 1 °C) to guarantee a stable temperature; sampling time was 20 min for each sample. Once sampling was finished, the fibre was withdrawn into the needle and transferred to the injection port of the GC-MS system. The desorption conditions were identical for all the samples. Furthermore, blanks were performed before each first SPME extraction, and randomly repeated during each series. Quantitative comparisons of relative peaks areas were performed between the same chemicals in the different samples.

### 4.3. Gas Chromatography Coupled with Mass Spectrometry (GC-MS) and Compounds Identification

Gas chromatography-electron impact mass spectrometry (GC-EIMS) analyses were performed with an Agilent 7890B gas chromatograph (Agilent Technologies Inc., Santa Clara, CA, USA) equipped with an Agilent HP-5MS (Agilent Technologies Inc., Santa Clara, CA, USA) capillary column (30 m × 0.25 mm; coating thickness 0.25 μm) and an Agilent 5977B single quadrupole mass detector (Agilent Technologies Inc., Santa Clara, CA, USA). The analytical conditions were as reported in Ascrizzi et al. (2017) [18]: injector and transfer line temperatures 220 and 240 °C, respectively; oven temperature programmed from 60 to 240 °C at 3 °C/min; carrier gas helium at 1 mL/min; split ratio 1:25. The acquisition parameters were as follows: full scan; scan range: 30–300 *m*/*z*; scan time: 1.0 s. Identification of the constituents was based on a comparison of the retention times with those of the authentic samples, comparing their linear retention indices relative to the series of *n*-hydrocarbons. Computer matching was also used against commercial [19,20] and laboratory-developed mass spectra library built up from pure substances and components of commercial essential oils of known composition and MS literature data [21,22,23,24,25].

### 4.4. Statistical Analyses

The statistical analyses were carried out with the JMP 13 Pro software package (SAS Institute, Cary, NC, USA). The covariance data matrix for the headspace complete volatile compositions was a 69 × 6 matrix (69 individual compounds × 6 samples = 414 data). The principal component analysis (PCA) was performed selecting the two highest principal components (PCs) obtained by the linear regressions operated on mean-centered, unscaled data; as an unsupervised method, this analysis aimed at reducing the dimensionality of the multivariate data of the matrix, whilst preserving most of the variance [26]. The chosen PC1 and PC2 cover 91.58% and 6.72% of the variance, respectively, for a total explained variance of 98.30%. The hierarchical cluster analysis (HCA) was performed using the Ward’s method. Both the hierarchical cluster and principal component analyses can be applied as unsupervised methods to observe groups of samples even without reference samples that can be used as a training set to establish the model [27]. The main compounds in the headspace compositions were processed by two-way ANOVA, to assess the significance of the species × whole/cut factors interaction. Means among the whole or cut form of the same species, as well as among the same form (whole or cut) over the three species were separated by Tukey’s b *post-hoc* test. *p* < 0.05 was used as significance level of differences between means.

## 5. Conclusions

The present botanical classification of the Chiana Valley (Italy) typical elephant garlic, known as “Aglione della Valdichiana”, identifies this species as a leek variety. The phytochemical evaluation of the spontaneous volatile emission of whole and cut cloves of this specimen, compared to those of leek and garlic samples, though, evidenced a larger proximity with garlic, rather than leek. Further studies are needed to assess whether: (i) this is the case of only “Aglione della Valdichiana” among the elephant garlics of different geographical origins, thus identifying it as an ecotype or (ii) if this pattern is shared in elephant garlic specimens from other geographical origins.

## Figures and Tables

**Table 1:** Complete compositions of the headspaces of both whole and cut samples of elephant garlic (*Allium ampeloprasum* var. *holmense* (Mill.) Asch. et Graebn), garlic (*A. sativum* L.) and leek (*A. ampeloprasum* L.).

Compounds	l.r.i. ^1^	Relative Abundance ± SD						Interaction Species × Whole/Cut ^2^	
		Elephant Garlic		Garlic		Leek			
		Whole	Cut	Whole	Cut	Whole	Cut	F	*p*-Value
**methyl thiirane ^2^**	637	0.8 ± 0.04 ^B;a^	4.6 ± 0.06 ^A;b^	0.5 ± 0.01 ^B;b^	7.9 ± 0.05 ^A;a^	- ^3;c^	- ^c^	25,363.06	<0.0001
(*E*)-1-methylthio-1-propene	681	-	0.4 ± 0.11	-	-	-	-		
allyl methyl sulfide	696	-	-	-	0.3 ± 0.04	-	-		
2-pentanol	703	-	-	0.2 ± 0.21	-	-	-		
(*E*)-2,3-dimethylthiirane	746	-	-	0.2 ± 0.01	-	-	-		
dimethyl disulfide	746	-	0.3 ± 0.04	-	-	-	-		
1-methylbutyl acetate	849	-	-	-	-	0.3 ± 0.05	-		
diallyl sulfide	866	0.2 ± 0.01	1.0 ± 0.03	0.2 ± 0.06	1.2 ± 0.15	-	-		
allyl propylsulfide	871	-	0.3 ± 0.13	-	-	-	-		
(*Z*)-allyl(prop-1-en-1-yl)sulfane	888	-	0.1 ± 0.16	-	-	-	-		
**methyl-2-propenyl disulfide**	920	1.4 ± 0.03 ^B;b^	11.9 ± 0.02 ^A;b^	2.1 ± 0.01 ^B;a^	14.5 ± 0.02 ^A;a^	- ^c^	- ^c^	161,252.0	<0.0001
methyl propyl disulfide	927	0.5 ± 0.01	1.4 ± 0.01	-	-	1.8 ± 0.18	1.7 ± 0.75		
(*E*)-methylpropenyl disulfide	940	-	0.4 ± 0.04	-	-	0.3 ± 0.04	0.1 ± 0.08		
6-methyl-5-hepten-2-one	985	0.1 ± 0.00	-	-	-	-	-		
myrcene	993	0.1 ± 0.00	-	-	-	-	-		
1,8-cineole	1034	1.3 ± 0.03	-	1.0 ± 0.01	-	-	-		
*n*-octanol	1071	2.7 ± 0.04	-	0.2 ± 0.01	-	-	-		
**diallyl disulfide**	1082	38.0 ± 0.74 ^B;b^	61.4 ± 0.16 ^A;b^	83.4 ± 0.30 ^A;a^	75.8 ± 0.11 ^B;a^	- ^c^	- ^c^	6839.732	<0.0001
**1-allyl-2-isopropyl disulfane**	1098	20.0 ± 0.35 ^A;a^	11.6 ± 0.03 ^B;a^	- ^b^	- ^c^	0.3 ± 0.03 ^C;b^	0.3 ± 0.08 ^C;b^	3136.357	<0.0001
**(*E*)-1-allyl-2-(prop-1-en-1-yl) disulfane**	1103	5.2 ± 0.06 ^A;a^	3.3 ± 0.01 ^B;a^	1.0 ± 0.00 ^A;b^	- ^B;b^	- ^c^	- ^b^	3031.800	<0.0001
nonanal	1104	0.6 ± 0.01	-	-	-	-	-		
(*Z*)-1-allyl-2-(prop-1-en-1-yl) disulfane	1107	-	-	0.2 ± 0.01	-	-	-		
**dipropyl disulfide**	1110	3.0 ± 0.05 ^A;b^	1.1 ± 0.01 ^B;b^	- ^c^	- ^b^	67.3 ± 2.52 ^B;a^	81.4 ± 6.33 ^A;a^	29.5631	<0.0001
**(*E*)-1-(prop-1-en-1-yl)-2-propyl disulfane**	1118	2.3 ± 0.15 ^A;b^	0.7 ± 0.01 ^B;b^	- ^c^	- ^b^	11.3 ± 2.01 ^A;a^	7.8 ± 4.11 ^A;a^	2.6408	0.1121
(*E*,*Z*)-di-1-propenyl disulfide	1124	-	-	-	-	0.4 ± 0.37	-		
(*E*,*E*)-propenyl disulfide	1129	0.1 ± 0.12	-	-	-	0.2 ± 0.32	0.3 ± 0.27		
methyl allyl trisulfide	1142	0.2 ± 0.05	0.4 ± 0.01	-	-	-	-		
methyl pentyl disulfide	1142	-	-	-	-	0.2 ± 0.29	0.1 ± 0.10		
camphor	1143	-	-	2.4 ± 0.00	-	-	-		
methylpropyl trisulfide	1150	-	-	-	-	0.6 ± 0.01	0.3 ± 0.13		
methyl (*E*)-propenyl trisulfide	1169	-	-	-	-	0.1 ± 0.20	-		
3-vinyl-1,2-dithiacyclohex-5-ene	1185	-	-	0.2 ± 0.01	-	-	-		
α-terpineol	1189	0.3 ± 0.11	-	-	-	-	-		
dihydro citronellol	1196	0.3 ± 0.07	-	-	-	-	-		
decanal	1204	0.5 ± 0.04	-	0.1 ± 0.07	-	0.4 ± 0.08	-		
verbenone	1205	0.2 ± 0.03	-	-	-	-	-		
2-vinyl-4H-1,3-dithiine	1206	-	-	0.9 ± 0.04	-	-	-		
*n*-decanol	1272	3.1 ± 0.13	-	-	-	-	-		
(*Z*)-3-tridecene	1284	0.1 ± 0.19	-	-	-	-	-		
(*E*)-3-tridecene	1285	0.1 ± 0.10	-	-	-	-	-		
2-undecanone	1294	0.1 ± 0.19	-	-	-	0.4 ± 0.10	-		
**diallyl trisulfide**	1297	2.0 ± 0.17 ^A;a^	0.7 ± 0.00 ^B;a^	0.7 ± 0.10 ^A;b^	- ^B;s^	- ^c^	- ^a^	185.4874	<0.0001
1-allyl-3-propyl trisulfane	1314	1.1 ± 0.13	0.2 ± 0.00	-	-	-	-		
**dipropyl trisulfide**	1328	0.5 ± 0.16 ^A;b^	- ^B;b^	- ^b^	- ^b^	5.9 ± 1.51 ^A;a^	5.3 ± 1.03 ^A;a^	0.5760	0.3093
(Z)-1-allyl-3-(prop-1-en-1-yl) trisulfane	1329	-	-	-	-	0.7 ± 0.01	-		
(E)-1-allyl-3-(prop-1-en-1-yl) trisulfane	1346	-	-	-	-	0.7 ± 0.06	0.7 ± 0.10		
3,5-diethyl-1,2,4-trithiolane	1352	-	-	-	-	1.7 ± 0.18	1.3 ± 0.11		
α-terpinyl acetate	1352	1.2 ± 0.02	-	0.5 ± 0.01	-	-	-		
cyclosativene	1368	-	-	-	-	0.1 ± 0.09	-		
α-copaene	1376	0.2 ± 0.04	-	-	-	-	-		
*n*-tetradecane	1400	-	-	-	-	0.2 ± 0.01	-		
β-caryophyllene	1420	0.4 ± 0.03	-	0.2 ± 0.01	-	0.1 ± 0.04	-		
β-copaene	1429	-	-	0.1 ± 0.00	-	-	-		
1-(1-(methylthio)propyl)-2-propyl disulfane	1431	-	-	-	-	0.1 ± 0.08	0.4 ± 0.52		
(*E*)-geranyl acetone	1455	0.4 ± 0.01	-	-	-	-	-		
valencene	1492	-	-	0.3 ± 0.04	-	-	-		
*n*-pentadecane	1500	0.1 ± 0.09	-	-	-	0.1 ± 0.08	-		
(*E*,*E*)-α-farnesene	1507	-	-	-	-	0.1 ± 0.20	-		
diallyl tetrasulfide	1540	-	0.2 ± 0.01	-	-	-	-		
caryophyllene oxide	1581	0.2 ± 0.06	-	-	-	-	-		
1-(1-(prop-1-en-1ylthio)propyl)-2-propyl disulfane	1592	-	-	-	-	-	0.4 ± 0.55		
***n*-hexadecane**	1600	10.8 ± 0.24 ^A;a^	- ^B^	5.5 ± 0.28 ^A;b^	- ^B^	0.1 ± 0.10 ^A;c^	- ^A^	3447.491	<0.0001
1-tetradecanol	1676	-	-	-	-	0.1 ± 0.11	-		
*n*-heptadecane	1700	-	-	-	-	0.1 ± 0.18	-		
octyl octanoate	1779	1.1 ± 0.02	-	-	-	-	-		
*n*-octadecene	1793	-	-	-	-	0.1 ± 0.01	-		
isopropyl tetradecanoate	1830	-	-	-	-	1.4 ± 0.78	-		
hexadecanal	1842	-	-	-	-	0.2 ± 0.01	-		
*iso*amyl dodecanoate	1846	0.7 ± 0.01	-	-	-	0.9 ± 0.55	-		
**Monoterpene hydrocarbons**		0.1 ± 0.00 ^A;a^	- ^B^	- ^b^	-	- ^b^	-	144.0000	<0.0001
**Oxygenated monoterpenes**		3.1 ± 0.20 ^A;b^	- ^B^	3.9 ± 0.01^A;a^	- ^B^	- ^c^	-	1938.579	<0.0001
**Sesquiterpene hydrocarbons**		0.6 ± 0.07 ^A;a^	- ^B^	0.6 ± 0.05 ^A;a^	- ^B^	0.3 ± 0.33 ^A;a^	- ^A^	3.2626	0.0739
**Oxygenated sesquiterpenes**		0.2 ± 0.06 ^A;a^	- ^B^	- ^b^	-	- ^b^	-	51.1579	<0.0001
**Apocarotenoids**		0.4 ± 0.01 ^A;a^	- ^B^	- ^b^	-	- ^b^	-	14161.00	<0.0001
**Sulfur compounds**		75.3 ± 0.42 ^B;c^	100.0 ± 0.01 ^A;a^	89.4 ± 0.23 ^B;b^	99.7 ± 0.00 ^A;b^	91.6 ± 0.54 ^B;a^	100 ± 0.01 ^A;a^	2678.860	<0.0001
**Other non-terpene derivatives**		20.0 ± 0.29 ^A;a^	- ^B^	5.9 ± 0.41 ^A;b^	- ^B^	4.4 ± 1.03 ^A;c^	- ^B^	1005.692	<0.0001
Total identified (%):		99.6 ± 0.07	100 ± 0.01	99.8 ± 0.24	99.7 ± 0.00	96.3 ± 0.16	100 ± 0.01		

^1^ Linear retention indices on a DB5 capillary column. ^2^ For compounds reported in bold and chemical classes, along the same row: (i) different superscript uppercase letters (A,B) indicate significant differences (Tukey’s HSD, *p* < 0.05) between the whole and cut form of the same species; ii) different superscript lowercase letters (a,b,c) indicate significant differences (Tukey’s HSD, *p* < 0.05) among the whole/cut form of the three species; (iii) the strength (F) and statistical significance (*p*-value) of the interaction between the two evaluated factors (species × whole/cut) are reported. ^3^ Not detected.
